# Sex-Based Impact of Creatine Supplementation on Depressive Symptoms, Brain Serotonin and SSRI Efficacy in an Animal Model of Treatment-Resistant Depression

**DOI:** 10.3390/ijms22158195

**Published:** 2021-07-30

**Authors:** Shami Kanekar, Robert Ettaro, Michael D. Hoffman, Hendrik J. Ombach, Jadeda Brown, Cayla Lynch, Chandni S. Sheth, Perry F. Renshaw

**Affiliations:** 1 Diagnostic Neuroimaging, Department of Psychiatry, University of Utah, Salt Lake City, UT 84108, USA; Robert.Ettaro@utah.edu (R.E.); michael.d.hoffman@hsc.utah.edu (M.D.H.); hendrik.ombach@gmail.com (H.J.O.); Jadeda.brown@hsc.utah.edu (J.B.); cjl00006@mix.wvu.edu (C.L.); chandni.sheth@utah.edu (C.S.S.); Perry.Renshaw@hsc.utah.edu (P.F.R.); 2 VISN19 MIRECC, 500 Foothill Drive, Salt Lake City, UT 84148, USA; 3 Veterans Affairs Salt Lake City Health Care System, 500 Foothill Drive, Salt Lake City, UT 84148, USA

**Keywords:** altitude, hypobaric hypoxia, brain bioenergetics, dietary creatine, serotonin, sex differences in depression, animal model of depression, SSRI efficacy, treatment-resistant depression, female depression

## Abstract

Background: Rates of major depressive disorder (MDD) increase with living at altitude. In our model, rats housed at moderate altitude (in hypobaric hypoxia) exhibit increased depression-like behavior, altered brain serotonin and a lack of antidepressant response to most selective serotonin reuptake inhibitors (SSRIs). A forebrain deficit in the bioenergetic marker creatine is noted in people living at altitude or with MDD. Methods: Rats housed at 4500 ft were given dietary creatine monohydrate (CRMH, 4% *w/w*, 5 weeks) vs. un-supplemented diet, and impact on depression-like behavior, brain bioenergetics, serotonin and SSRI efficacy assessed. Results: CRMH significantly improved brain creatine in a sex-based manner. At altitude, CRMH increased serotonin levels in the female prefrontal cortex and striatum but reduced male striatal and hippocampal serotonin. Dietary CRMH was antidepressant in the forced swim test and anti-anhedonic in the sucrose preference test in only females at altitude, with motor behavior unchanged. CRMH improved fluoxetine efficacy (20 mg/kg) in only males at altitude: CRMH + SSRI significantly improved male striatal creatine and serotonin vs. CRMH alone. Conclusions: Dietary CRMH exhibits sex-based efficacy in resolving altitude-related deficits in brain biomarkers, depression-like behavior and SSRI efficacy, and may be effective clinically for SSRI-resistant depression at altitude. This is the first study to link CRMH treatment to improving brain serotonin.

## 1. Introduction

Major depressive disorder (MDD) is a mental health disorder characterized by low mood, which is often chronic and can significantly diminish quality of life. MDD affects ~7% of adults and 13% of adolescents in the US, and the lifetime prevalence of MDD is 17% [1,2]. Almost a third of MDD patients worldwide fail to respond to the current standard-of-care antidepressants, and treatment-resistant depression (TRD) markedly worsens MDD burden [3]. Both MDD and TRD are linked to suicidal ideation and suicidal behavior. Rates of MDD and suicide both increase with altitude of residence [4,5,6,7,8,9,10,11,12,13,14], and people at altitude are exposed to hypobaric hypoxia (low partial pressure of oxygen). A similar pattern of increased rates of MDD and suicide is seen in people exposed to chronic hypoxia via disorders such as asthma, chronic obstructive pulmonary disorder (COPD), chronic bronchitis, cardiovascular disease and smoking [15,16,17,18,19,20]. Chronic hypoxia exposure has thus been proposed to increase risk for MDD and suicide [21,22].

Living at moderate altitude can alter blood oxygen saturation: arterial blood oxygen levels are distinctly lower in healthy people residing at 4500 ft vs. those at sea level [23]. The human brain makes up only 2% of body volume but at rest uses ~20% of total energy consumed by the body, and human brain physiology may be altered by the oxygen deficit at moderate altitude. Live neuroimaging of healthy long-term residents at 4500 ft vs. those at sea level shows changes in brain intracellular pH, suggesting that brain homeostasis is altered at moderate altitude [24]. Additionally, in a neuroimaging study of age- and gender-matched healthy long-term residents of Salt Lake City (at 4500 ft) vs. residents of Belmont, MA or Charleston, SC (at 20 ft), those at altitude exhibit markedly reduced forebrain levels of the bioenergetic marker creatine vs. those at sea level [25]. A similar pattern of reduced brain creatine is also noted in neuroimaging studies of rats after housing at altitude [26,27]. The hypoxia experienced with living at moderate altitude may thus lead to brain metabolic dysfunction.

MDD is linked to brain hypometabolism, with the severity of MDD directly linked to that of bioenergetic deficit [28,29,30]. Excitable cells in both the brain and muscle depend on a constant supply of vast amounts of energy for normal function, and the creatine pathway plays a complex role in cell energy homeostasis in both [31]. Cellular creatine is phosphorylated by the enzyme creatine kinase to form phosphocreatine (PCR), a high-energy phosphate generator (or phosphagen). In the brain, cellular creatine/PCR levels directly impact the available levels of the key energy currency, ATP (adenosine triphosphate) [29]. ATP is synthesized in mitochondria by oxidative phosphorylation in a time-intensive process, and the high-energy phosphate generated is transported to the cytosol by the creatine/PCR pathway. The brain’s fluctuating high energy needs call for quick, reliable renewal of cellular ATP, and cellular PCR provides a high energy phosphate for rapid regeneration of ATP from ADP (adenosine diphosphate) in the cell [29]. The ATP regeneration capacity of the creatine/PCR pathway is very high compared to other modes of ATP synthesis: in muscle, the creatine/PCR pathway is 12 times faster at restoring cell ATP levels vs. mitochondrial ATP synthesis and 75 times faster than de novo synthesis [31]. The creatine/PCR pathway thus forms a vital cell energy buffering system needed for effective brain function, which may be especially critical in hypoxia-induced hypometabolism.

Creatine is mostly acquired by dietary consumption, and the primary source of dietary creatine is animal-based protein. When US national health and nutritional data were analyzed for creatine intake, an inverse relationship was found between creatine intake and depression status. People in the highest quartile of creatine intake had an average depression rate of 6% vs. 10.3% in those in the lowest quartile [32]. The link between dietary creatine intake and depression was strongest in women, in people aged 29–64 and those not using antidepressants. When tested clinically, treatment with oral creatine monohydrate (CRMH) was found to improve forebrain creatine in healthy volunteers [33,34] and to enhance the antidepressant efficacy of selective serotonin reuptake inhibitors (SSRIs) in patients with MDD [35,36,37]. For treatment with CRMH alone, oral doses ranged from 0.3 g/kg/day for 2 weeks [34] to 4 × 5 g/day for 4 weeks [35], and these studies were conducted solely in healthy male volunteers [34] or in both sexes with no sex-based stratification [35]. CRMH augmentation of SSRI function has been studied solely in women MDD patients, with doses ranging from 2 to 10 g/day for 8 weeks [36,37,38]. Similar beneficial effects of oral CRMH have been noted in sex-based preclinical studies conducted in rats at sea level [38,39]. CRMH supplementation (4% *w/w*, 5 weeks) showed a trend to improve depression-like behavior in the FST in female Sprague Dawley (SD) rats at sea level but was depressant in males [39]. Additionally, oral CRMH treatment improved efficacy of a sub-acute dose of the SSRI fluoxetine (5 mg/kg) in the FST in female rats but not in males [40]. The impact of dietary CRMH treatment on rodent brain creatine or serotonin levels was not measured in these studies. Both preclinical and clinical studies thus show oral CRMH treatment to be beneficial for depressive symptoms in females.

We established an animal model to study the impact of living at moderate altitude on depression-like behavior. We found that Sprague Dawley rats housed at moderate altitude (4500 ft, 10,000 ft) exhibit more depression-like behavior in the forced swim test (FST) and anhedonia in the sucrose preference test (SPT) vs. those at sea level, with sex-based differences [40,41]. Rats housed at moderate altitude also exhibit a sex-based disruption in brain serotonin [41], and forebrain deficits in brain energetic markers [27]. In female rats, a sustained decrease in brain serotonin and increase in depression-like behavior is seen with housing at moderate altitude, while males exhibit a transient increase in both regional brain serotonin and depression-like behavior [41]. A serotonin deficiency is linked to SSRI lack of efficacy in the rodent and mouse FST. Despite this, both male and female rats at altitude exhibit a sustained lack of antidepressant response to most SSRIs, including fluoxetine (Prozac^®^), escitalopram (Lexapro^®^) and paroxetine (Paxil^®^) in the FST [42]. SSRIs make up over 80% of antidepressants prescribed in the US; thus, loss of SSRI function may render MDD more chronic and persistent at altitude.

Since living at altitude is linked to brain hypometabolism, we asked whether dietary CRMH might be functional as an antidepressant for SSRI-resistant depression at moderate altitude. Our hypothesis was that CRMH treatment may improve rodent brain energetic function in regions linked to MDD and may have antidepressant efficacy in rats at altitude. In the current study, we first describe the sex-based impacts of duration at altitude on creatine levels in brain regions involved in depression-like behavior, and then conduct a sex-based study on use of CRMH treatment for depression-like behavior at altitude, examining impact on behavior, brain chemistry and SSRI function.

## 2. Results

### 2.1. Impact of Duration at Altitude on Brain Creatine

**Figure 1:**
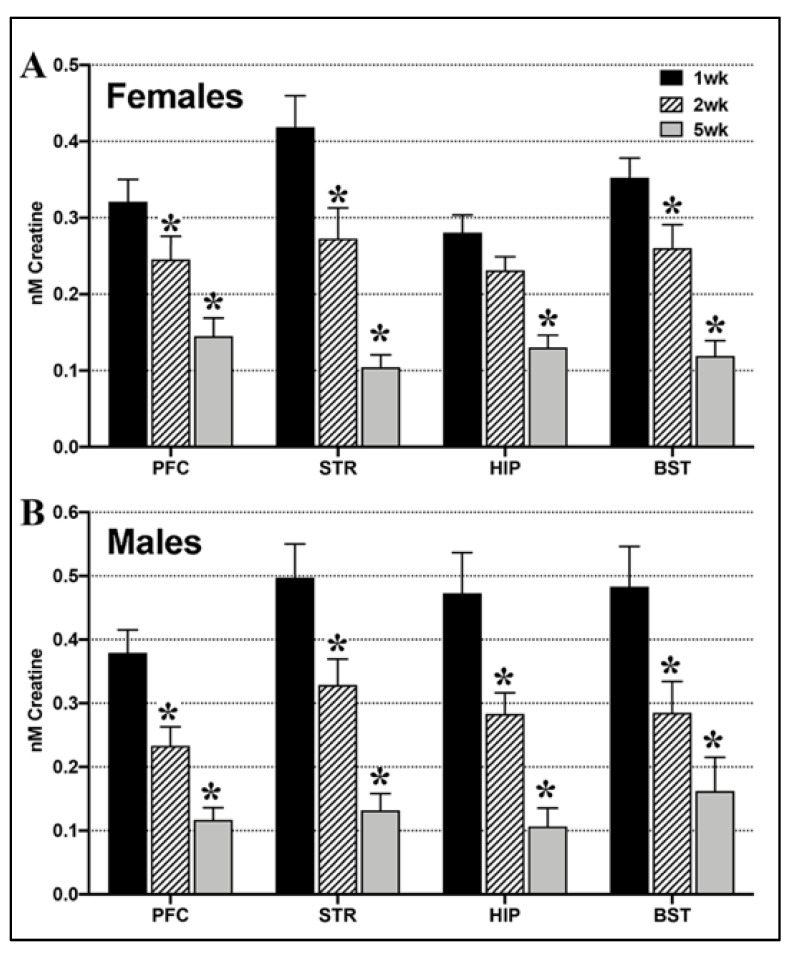
Brain creatine levels decrease with duration at moderate altitude in both sexes. (**A**). In females, brain creatine decreased from 1 week at altitude (4500 ft) to be significantly lower at 2 weeks and 5 weeks in the PFC, STR, HIP and BST. (**B**). In males, brain creatine also decreased from 1 week to be lower at 2 weeks and 5 weeks at altitude in all 4 brain regions. (One-way ANOVA, * = *p* < 0.05 vs. 1 week).

Endogenous brain creatine levels were compared after housing rats at altitude (4500 ft) for 1–5 weeks.

(A) Females: One-way ANOVA showed a significant effect of time at altitude on endogenous creatine in the female PFC (F_2,37_ = 8.13, *p* = 0.001), STR (F_2,35_ = 11.89, *p* = 0.0001), HIP (F_2,39_ = 8.64, *p* = 0.0008) and BST (F_2,41_ = 11.45, *p* = 0.0001, Figure 1A). Post hoc tests showed that creatine levels in the female PFC, STR and BST were significantly lower at 2 weeks and 5 weeks vs. 1 week at altitude, while HIP creatine levels were significantly lower at 5 weeks vs. 1 week (*p* < 0.05).

(B) Males: Endogenous brain creatine levels also decreased significantly with time at altitude in the male PFC (F_2,40_ = 11.45, *p* = 0.0001), STR (F_2,40_ = 9.59, *p* = 0.0004), HIP (F_2,41_ = 7.78, *p* = 0.001), and BST (F_2,41_ = 5.72, *p* = 0.006, Figure 1B). In post-hoc tests, male endogenous creatine levels were significantly lower at both 2 weeks and 5 weeks vs. baseline in all 4 brain regions (*p* < 0.05).

### 2.2. Dietary CRMH Supplementation: CRMH Dosage and Impact on Body Weight

Rats were supplemented with dietary CRMH in two groups: the dietary CRMH group (CRDS study) to study the impact of dietary CRMH on brain chemistry and behavior, and the dietary CRMH and SSRI group (CRDS + SSRI study) to study the impact of dietary CRMH on SSRI efficacy at altitude. The CRDS and CRDS + SSRI groups were compared here for food consumed, body weights and CRMH dosage.

#### 2.2.1. Food Consumed

**Figure 2:**
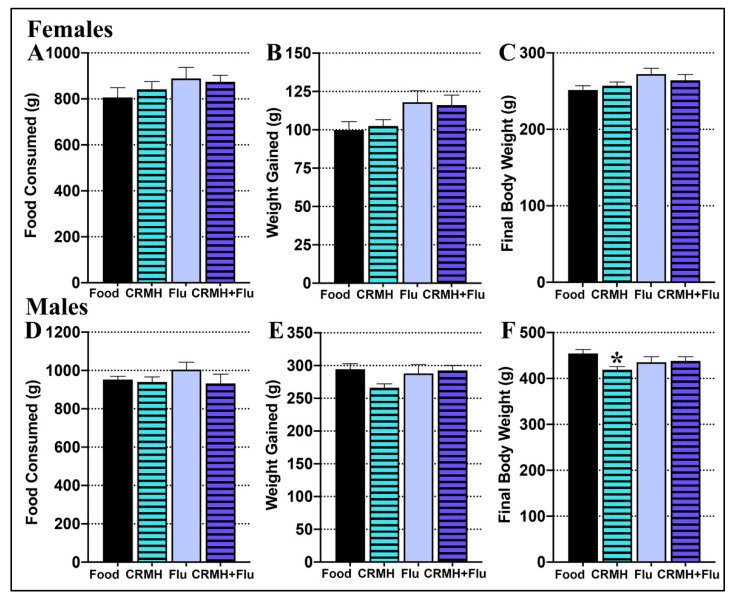
Food consumed, body weight gain and final body weight after 5 weeks of CRMH supplementation at altitude. Females in the food control and dietary CRMH groups for the CRDS and CRDS + SSRI studies show no difference in (**A**). food consumed over 5 weeks, (**B**). total weight gained or (**C**). final body weight. Males show no difference between the dietary groups in (**D**). food consumed over 5 weeks, (**E**). total weight gained or (**F**). final body weight, * = *p* < 0.05.

(A) Females: In the CRDS study, females in the food group (*n* = 15) consumed an average of 807 ± 42 gms, while those in the CRMH group (*n* = 18) consumed a total of 842 ± 32 gms. In the CRDS + SSRI study, females in the food group (*n* = 15) consumed a total of 889 ± 49 gms over 5 weeks, while those in the CRMH group (*n* = 20) consumed 875 ± 28 gms. One-way ANOVA showed no difference in food consumed between groups over the 5 weeks period (F_3,56_ = 0.889, *p* = 0.45, Figure 2A).

(B) Males: For the CRDS study, males in the food group (*n* = 15) consumed 957 ± 17 gms, while those in the CRMH group (*n* = 19) consumed 940 ± 27 gms. In the CRDS + SSRI study, males in the food group (*n* = 11) consumed 1005 ± 38 gms, while those in the CRMH group (*n* = 13) consumed 933 ± 48 gms of food over 5 weeks of treatment. One-way ANOVA showed no difference between the four male groups in total food consumed (F_3,54_ = 0.842, *p* = 0.47, Figure 2D).

#### 2.2.2. Body Weight Gain

(A) Females: Females in the CRDS study had an average weight gain of 100 ± 5 gms for the food group and 102 ± 4 gms for the CRMH group over 5 weeks of treatment. In the CRDS + SSRI study, females had an average weight gain of 118 ± 7 gms for the food group and 116 ± 7 gms for the CRMH group. One-way ANOVA showed that female groups did not vary in weight gained over 5 weeks of treatment (F_3,59_ = 2.41, *p* = 0.076, Figure 2B).

(B) Males: Males in the CRDS study had a weight gain of 299 ± 9 gms for the food group and 266 ± 6 gms for the CRMH group after 5 weeks of treatment. In the CRDS + SSRI study, males gained 288 ± 13 gms in the food group and 293 ± 8 gms in the CRMH group over 5 weeks. In one-way ANOVA, males varied in weight gain over 5 weeks (F_3,52_ = 2.83, *p* = 0.047, Figure 2E), but post hoc tests showed no difference between groups.

#### 2.2.3. Final Body Weight

(A) Females: In females in the CRDS study, final body weight after 5 weeks of treatment was 251 ± 6 gms for the food group and 257 ± 5 gms for the CRMH group. For females in the CRDS + SSRI study, the final body weight was 272 ± 8 gms for the food group and 264 ± 8 gms for the CRMH group. In one-way ANOVA, the final body weight did not differ between female groups (F_3,56_ = 1.85, *p* = 0.14, Figure 2C).

(B) Males: In the CRDS study, the final body weight at 5 weeks was 455 ± 8 gms for the male food group and 419 ± 7 gms for the CRMH group. For the CRDS + SSRI study, the final body weight was 435 ± 12 gms for the food group and 438 ± 9 gms for the CRMH group. In one-way ANOVA, male body weight varied by group (F_3,53_ = 3.24, *p* = 0.03, Figure 2F), with the CRDS CRMH group significantly lower than the CRDS food group (*p* < 0.05).

#### 2.2.4. Creatine Consumption

**Figure 3:**
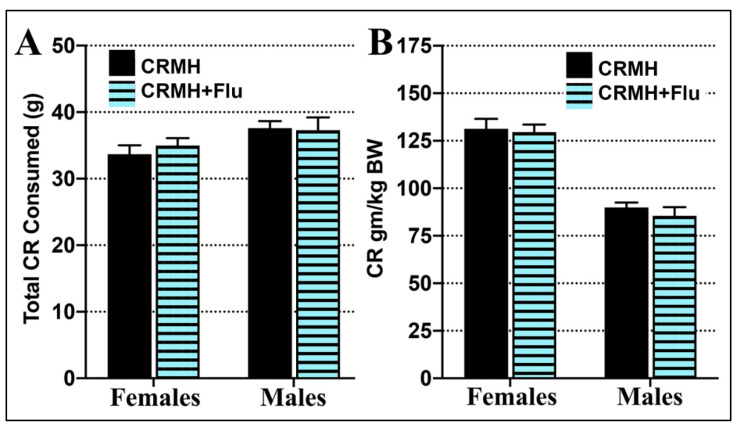
Total CRMH consumed over 5 weeks of supplementation at altitude. Both females and males in the dietary supplementation groups for the CRDS or CRDS + SSRI studies show no difference in (**A**). total CRMH consumed or (**B**). CRMH consumed as gm/kg body weight.

(A) Females: In females, total CRMH consumption did not vary significantly between CRDS (33.6 ± 1 gms) and CRDS + SSRI (35.2 ± 1 gms) groups over 5 weeks (Student’s *t*-test, *p* = 0.47, Figure 3A). In females, CRMH dosage (CR consumed in gm/kg final body weight) also did not vary between the CRDS (131.3 ± 6 gm/kg) and CRDS + SSRI groups (129.5 ± 6 gm/kg, *p* = 0.79, Figure 3A).

(B) Males: Similarly, male CRMH consumption did not vary significantly between CRDS (37.6 ± 1 gms) and CRDS + SSRI (37.3 ± 2 gms) groups over 5 weeks (*p* = 0.89, Figure 3A). Male CRMH dosage also did not differ between CRDS (89.9 ± 3 gm/kg) and CRDS + SSRI groups (85.5 ± 5 gm/kg, *p* = 0.79, Figure 3B).

### 2.3. Creatine Monohydrate Dietary Supplementation (CRDS) Study

The impact of dietary CRMH was assessed on brain energetics, brain serotonin and behavior in rats housed at moderate altitude.

#### 2.3.1. CRDS Impact on Blood and Brain Creatine

**Figure 4:**
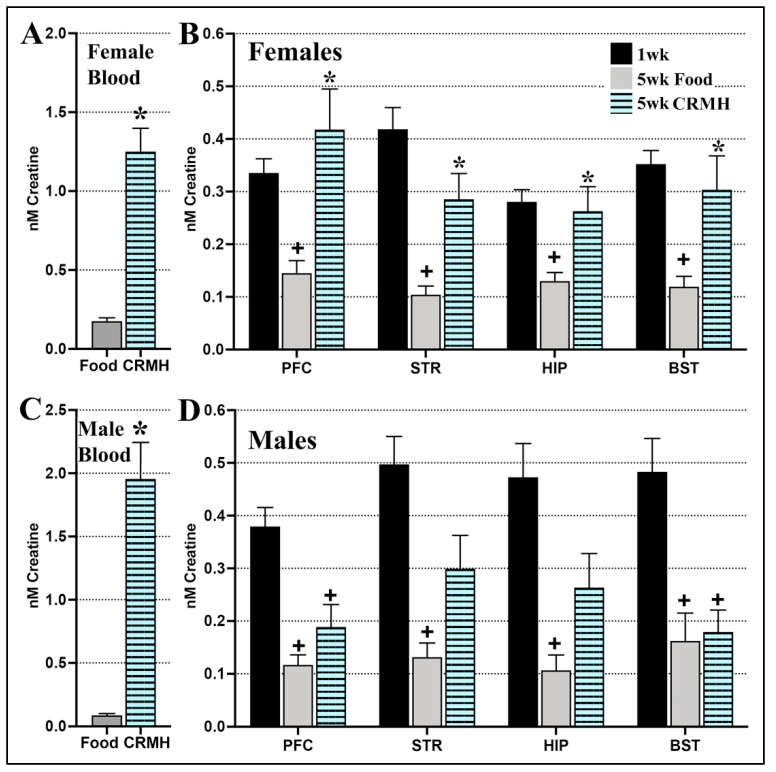
Dietary CRMH significantly increases creatine levels in the blood and brain of both sexes at altitude. (**A**). In females at altitude, dietary CRMH significantly increased blood creatine levels vs. food alone. (**B**). In females, CRMH augmentation significantly increased creatine in the PFC, STR, HIP and BST vs. food controls. Dietary CRMH was effective at resolving the drop in female brain creatine seen from 1 week to 5 weeks at altitude. (**C**). In males at altitude, dietary CRMH increased blood creatine levels vs. food alone. (**D**). In males, oral CRMH significantly increased creatine in the STR and HIP vs. food controls (Student’s *t*-test), but not in the PFC or BST. CRMH supplementation did not resolve the altitude-related brain creatine deficit in males. (One-way ANOVA, * = *p* < 0.05 vs. food controls, + = *p* < 0.05 vs. 1 week altitude or baseline).

(A) Females: (a) Blood creatine levels: Female blood creatine levels increased significantly with dietary CRMH supplementation vs. food (Student’s *t*-test, *p* < 0.0001, *n* = 11–15, Figure 4A). (b) Brain creatine levels: Female brain creatine levels also increased significantly with dietary CRMH in the PFC (Student’s *t*-test, *p* = 0.01), STR (0.009), HIP (*p* = 0.04) and BST (*p* = 0.03, Figure 4B). When the food and CRMH groups were compared to the 1 week group (baseline), one-way ANOVA showed a significant effect of CRDS on improving brain creatine in all four regions to baseline levels: PFC (F_2,34_ = 6.12, *p* = 0.0054), STR (F_2,36_ = 11.52, *p* = 0.0001), HIP (F_2,35_ = 4.73, *p* = 0.01) and the BST (F_2,37_ = 7.44, *p* = 0.002, Figure 4B). Post hoc tests showed that in all four regions, the female food group was significantly lower in creatine vs. both baseline and dietary CRMH groups (*p* < 0.05), while the dietary CRMH and 1 week groups were not significantly different from each other.

(B) Males: (a) Blood creatine levels: In males, blood creatine rose significantly with dietary CRMH (Student’s *t*-test, *p* < 0.0001, *n* = 11–13, Figure 4C). (b) Brain creatine levels: Male brain creatine levels increased significantly with CRMH treatment vs. food in the STR (Student’s *t*-test, *p* = 0.03) and HIP (*p* = 0.04), but not in the PFC (*p* = 0.23) or BST (*p* = 0.18, Figure 4D). When the food and CRMH groups were compared to the 1 week altitude group, one-way ANOVA showed a significant effect on brain creatine levels in the PFC (F_2,37_ = 11.43, *p* = 0.0001), STR (F_2,34_ = 8.56, *p* = 0.001) and HIP (F_2,35_ = 6.43, *p* = 0.004), and the BST (F_2,37_ = 8.2, *p* = 0.001, Figure 4D). In post hoc tests, both the food and CRMH groups were significantly lower vs. baseline in the male PFC and BST (*p* < 0.05), while the food group was significantly lower than baseline in the STR and HIP.

#### 2.3.2. CRDS Impact on Brain Serotonin

**Figure 5:**
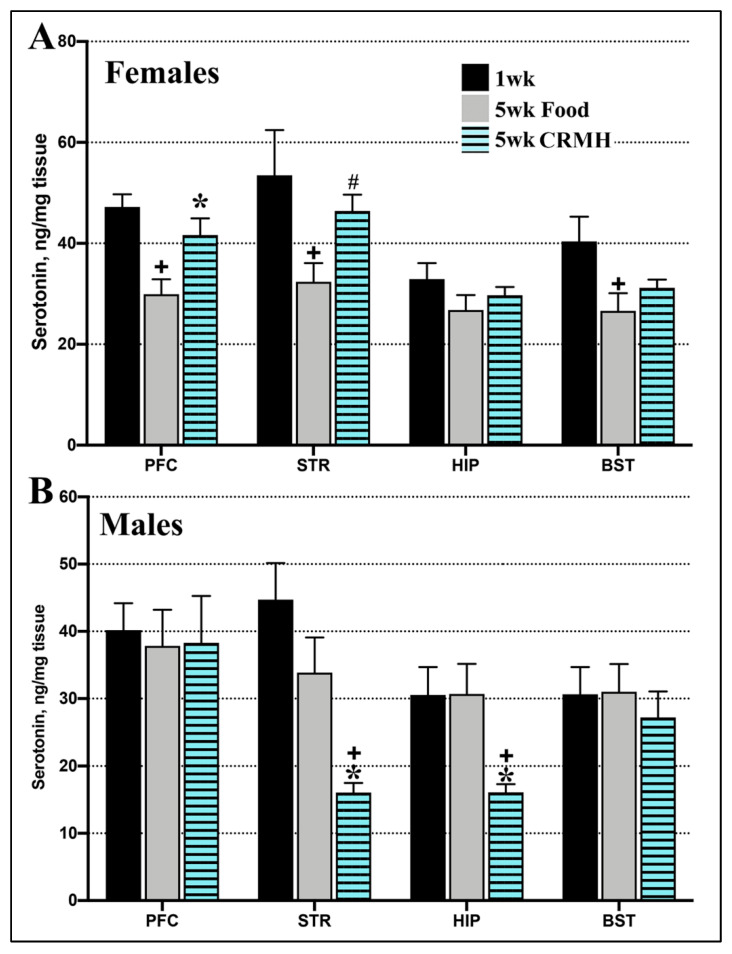
Dietary CRMH significantly increases brain serotonin levels in female rats at altitude but reduces brain serotonin in males at altitude. (**A**). In females, CRDS significantly increased serotonin levels in the PFC and STR vs. food controls but did not significantly change serotonin levels in the HIP or BST. In the female PFC and STR, the CRMH group was not significantly different from the 1 week altitude group. (**B**). In males, CRMH treatment significantly decreased serotonin in the STR and HIP, with no effects in the PFC or BST (one-way ANOVA, * = *p* < 0.05 vs. food controls, + = *p* < 0.05 vs. 1 week altitude or baseline, # = *p* < 0.05).

(A) Females: In females, dietary CRMH treatment improved the altitude-related deficit in brain serotonin in the PFC (Student’s *t*-test, *p* = 0.01, *n* = 16–19) and STR (*p* = 0.008, Figure 5A). Dietary CRMH did not significantly alter serotonin in the HIP (*p* = 0.41) or BST (*p* = 0.26) vs. controls. When food and CRMH groups were compared to the baseline (1 week) group, one-way ANOVA showed a significant effect in the PFC (F_2,38_ = 7.41, *p* = 0.0019), STR (F_2,41_ = 4.58, *p* = 0.02) and BST (F_2,43_ = 3.92, *p* = 0.03), but not the HIP (F_2,43_ = 1.18, *p* = 0.32). In the PFC, STR and BST, post hoc tests showed that food groups has significantly lower serotonin levels vs. baseline (1 week at 4500 ft). In the PFC, CRMH treatment significantly increased serotonin vs. controls (*p* < 0.05), while in both the PFC and STR, the CRMH group was not significantly different from baseline.

(B) Males: In contrast, dietary CRMH significantly reduced serotonin in the male STR (Student’s *t*-test, *p* = 0.005, *n* = 13–17) and HIP (*p* = 0.002, Figure 5B) vs. controls, but did not alter serotonin in the PFC (*p* = 0.57) or BST (*p* = 0.51). When the food and CRMH groups were compared to the baseline group, one-way ANOVA showed a significant effect in the STR (F_2,32_ = 9.69, *p* = 0.0005) and HIP (F_2,32_ = 6.86, *p* = 0.003) but not in the PFC (F_2,36_ = 0.378, *p* = 0.687) or BST (F_2,38_ = 0.273, *p* = 0.76). Post hoc tests showed that CRMH treatment significantly reduced serotonin in the male STR and HIP vs. both the food and baseline groups.

#### 2.3.3. CRDS Impact on Behavior in the Sucrose Preference Test

**Figure 6:**
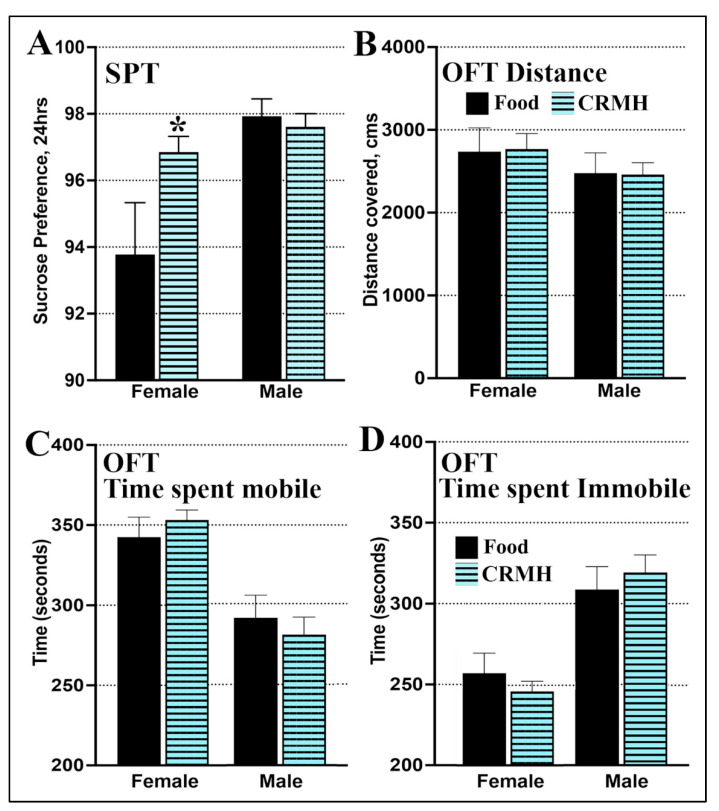
Dietary CRMH reduces anhedonia in the sucrose preference in only females at altitude and does not alter motor behavior in the open field test in either sex. (**A**). Females at altitude showed an increase in sucrose preference after dietary CRMH treatment vs. food, while males at altitude showed no difference between food and CRMH groups in the SPT. For OFT behavior, both females and males at altitude did not vary in (**B**). total distance covered in the OFT, (**C**). time spent mobile in the OFT or (**D**). time spent immobile in the OFT. (Student’s *t*-test, * = *p* < 0.05 vs. food controls).

(A) Females: For females at altitude, sucrose preference was significantly higher in the CRMH group vs. food controls (Student’s *t*-test, *p* = 0.04, *n* = 15–18, Figure 6A).

(B) Males: For males at altitude, sucrose preference did not change significantly with dietary CRMH treatment: the CRMH group was similar to food controls (*p* = 0.56, *n* = 15–18, Figure 6A).

#### 2.3.4. CRDS Impact on Behavior in the Open Field Test

A separate set of animals were given dietary CRMH vs. food and tested in the OFT.

(A) Females: Females in the food group covered a total distance of 2737 ± 287 cm, while females in the CRMH group covered 2769 ± 187 cm. In females, distance covered thus did not differ between CRMH and control groups (Student’s *t*-test, *p* = 0.93, *n* = 16 (food group), *n* = 41 (CR group), Figure 6B). Females in the CRMH and food groups did not differ in time spent mobile (Figure 6C) or immobile (Figure 6D) in the OFT (*p* = 0.40).

(B) Males: Males in the food group covered a distance of 2478 ± 244 cm while the CRMH group covered 2461 ± 143 cm. Male distance covered did not differ between groups (*p* = 0.95, *n* = 13 (food group), *n* = 34 (CR group), Figure 6B). Males in the CRMH group also did not vary from food controls in time spent mobile (Figure 6C) or immobile (*p* = 0.62, Figure 6D).

#### 2.3.5. CRDS Impact on Behavior in the Forced Swim Test

**Figure 7:**
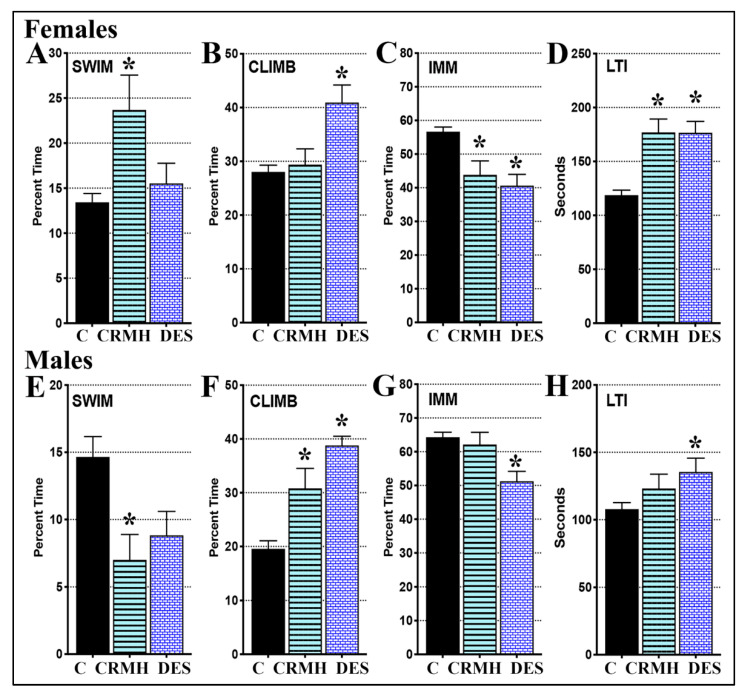
Dietary CRMH has antidepressant effects in the forced swim test in female rats at altitude but not males. In females at altitude, dietary CRMH significantly (**A**). increased swimming, (**B**). did not change climbing, (**C**). decreased immobility and (**D**). increased LTI vs. controls. Antidepressant efficacy of dietary CRMH in females was on par with that of the TCS desipramine (DES). In males at altitude, dietary CRMH significantly (**E**). decreased swimming, (**F**). increased climbing, and did not alter (**G**). immobility or (**H**). LTI (one-way ANOVA, * = *p* < 0.05 vs. controls).

(A) Females: For females, one-way ANOVA showed a significant impact of CRMH treatment on swimming (F_2,94_ = 7.09, *p* = 0.0014), climbing (F_2,100_ = 8.38, *p* = 0.0004), immobility (F_2,100_ = 13.85, *p* < 0.0001) and latency to immobility (LTI, F_2,97_ = 21.52, *p* < 0.0001, Figure 7A–D). Post hoc tests show that dietary CRMH significantly increased female swimming (*p* < 0.001), reduced immobility (*p* < 0.01) and increased LTI (*p* < 0.0001) vs. controls. The TCA desipramine significantly increased climbing (*p* < 0.001), decreased immobility (*p* < 0.0001) and increased LTI (*p* < 0.0001) vs. controls. In females, CRMH treatment reduced immobility by over 20%, and was as effective an antidepressant at altitude as desipramine.

(B) Males: For males, one-way ANOVA showed a significant impact of treatment on swimming (F_2,67_ = 3.735, *p* = 0.029), climbing (F_2,68_ = 16.25, *p* < 0.0001), immobility (F_2,71_ = 7.632, *p* = 0.001), and LTI (F_2,71_ = 3.412, *p* = 0.039, Figure 7E–H). Post hoc tests show that dietary CRMH significantly increased climbing (*p* < 0.01) but both immobility and LTI in CRMH groups were not significantly different from controls. Dietary CRMH also showed a trend to worsen swimming in males (Student’s *t*-test, *p* = 0.03). Desipramine significantly increased male climbing (*p* < 0.0001), reduced immobility (*p* < 0.001) and increased LTI (*p* < 0.05).

### 2.4. CRDS + SSRI Study

In rats housed at moderate altitude, the SSRI fluoxetine loses efficacy in both sexes. Dietary CRMH was evaluated for the impact on antidepressant efficacy of the SSRI fluoxetine in rats housed at moderate altitude.

#### 2.4.1. CRDS Impact on SSRI Function in the Forced Swim Test

**Figure 8:**
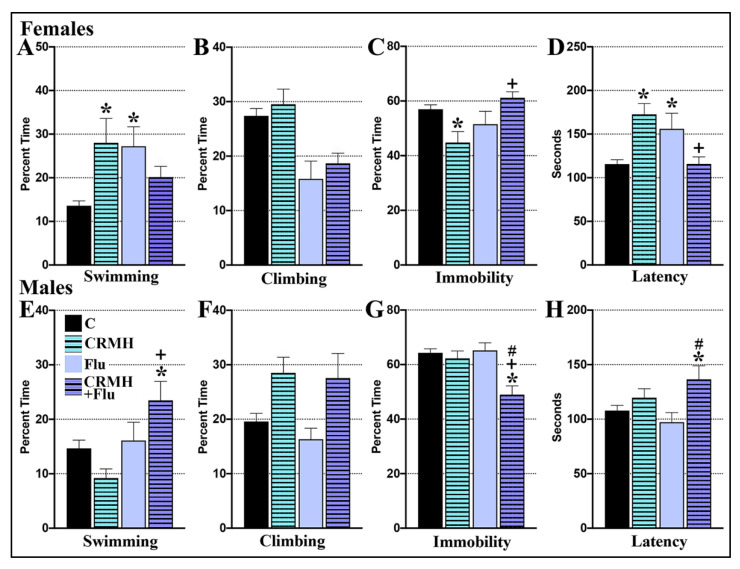
Dietary CRMH improves efficacy of the SSRI fluoxetine in male rats at altitude but not females. In females at altitude, CRMH + FLU did not significantly improve (**A**). swimming or (**B**). climbing behavior, and (**C**). significantly increased immobility and (**D**). decreased LTI vs. controls. In males at altitude, CRMH + FLU significantly (**E**). increased swimming, (**F**). did not significantly alter climbing, (**G**). decreased immobility and (**H**). increased LTI vs. controls (two-way ANOVA, * = *p* < 0.05 vs. food controls, + = *p* < 0.05 vs. dietary CR, # = *p* < 0.05 vs. FLU).

(A) Females: For female swimming, two-way ANOVA showed no main effects of dietary CRMH (F_1,97_ = 1.49, *p* = 0.22) or of the SSRI fluoxetine (FLU, F_1,97_ = 0.923, *p* = 0.33), but a significant effect was seen of their interaction (F_1,97_ = 12.74, *p* = 0.0006, Figure 8A). For female climbing, a significant effect was seen for SSRI alone (F_1,102_ = 20.91, *p* < 0.0001), but not dietary CRMH (F_1,102_ = 1.029, *p* = 0.31) or their interaction (F_1,102_ = 0.021, *p* = 0.88, Figure 8B). For immobility, no main effect was seen for dietary CRMH (F_1,102_ = 0.16, *p* = 0.68) or SSRI alone (F_1,102_ = 3.14, *p* = 0.079), but a significant effect was seen for their interaction (F_1,102_ = 12.71, *p* = 0.0006, Figure 8C). Similarly, for LTI, no main effects were seen of dietary CRMH (F_1,99_ = 0.71, *p* = 0.4) or SSRI (F_1,99_ = 0.68, *p* = 0.4) but a significant effect was seen of the interaction (F_1,99_ = 23.74, *p* < 0.0001, Figure 8D). In post hoc tests, both CRMH and fluoxetine each improved female swimming vs. controls (*p* < 0.05), but CRMH + FLU was not significantly different from controls (Figure 8A). In post hoc tests, CRMH significantly reduced female immobility (Figure 8C) and increased LTI (Figure 8D) vs. controls (*p* < 0.05). However, CRMH + FLU increased immobility and decreased LTI vs. CRMH and was not significantly different from controls for both immobility and LTI (Figure 8C,D).

(B) Males: Two-way ANOVA showed no effect of dietary CRMH (F_1,78_ = 0.144, *p* = 0.7) on male swimming, but significant effects were seen for SSRI alone (F_1,78_ = 9.75, *p* = 0.0025) and their interaction (F_1,78_ = 6.5, *p* = 0.01, Figure 8E). For climbing, a main effect was seen for dietary CRMH (F_1,80_ = 12.45, *p* = 0.0007) but not for SSRI (F_1,80_ = 0.535, *p* = 0.46) or their interaction (F_1,80_ = 0.162, *p* = 0.68, Figure 8F). For immobility, significant effects were seen for dietary CRMH (F_1,81_ = 12.25, *p* = 0.0008), SSRI (F_1,81_ = 5.72, *p* = 0.02) and their interaction (F_1,81_ = 7.399, *p* = 0.008, Figure 8G). For male LTI, a main effect was seen of CRMH alone (F_1,80_ = 8.38, *p* = 0.005) but none were seen for SSRI (F_1,80_ = 0.119, *p* = 0.73) or their interaction (F_1,80_ = 2.42, *p* = 0.12, Figure 8H). In post hoc tests, CRMH + FLU improved male swimming vs. both controls and dietary CR and decreased male immobility vs. all three of the other groups. For male LTI, CRMH + FLU significantly increased LTI in males vs. controls by Student’s *t*-test (*p* = 0.018), but these effects were lost in multiple comparisons.

#### 2.4.2. CRDS + SSRI Impact on Brain Creatine

**Figure 9:**
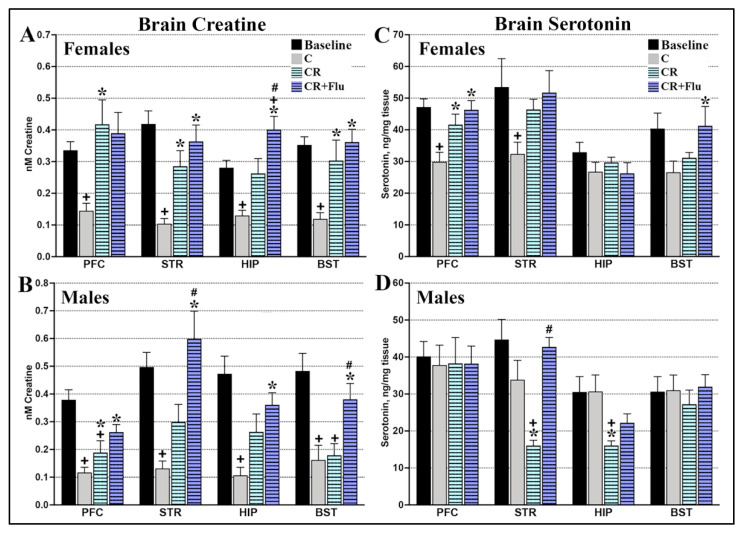
Dietary CRMH combined with SSRI alters brain creatine and serotonin vs. dietary CRMH alone. (1) Brain creatine: (**A**). in females at altitude, CRMH + FLU increased brain creatine vs. controls in all 4 brain regions but did not change regional brain creatine vs. dietary CRMH alone, except in the HIP. (**B**). In males, CRMH + FLU also increased brain creatine levels vs. controls in all 4 regions. CRMH + FLU also increased striatal brain creatine levels vs. dietary CRMH alone, to resolve the deficit in brain creatine seen after 5 weeks at altitude. (2) Brain serotonin: (**C**). in female rats at altitude, CRMH + FLU significantly increased serotonin in the PFC, STR and BST vs. food controls, but did not alter regional serotonin in any region vs. dietary CRMH alone. The female HIP was unchanged with treatment. (**D**). In males, CRMH + FLU improved serotonin in the STR vs. dietary CRMH alone but did not significantly alter serotonin levels in the other 3 regions. One-way ANOVA, * = *p* < 0.05 vs. food controls, # = *p* < 0.05 vs. dietary CR, + = *p* < 0.05 vs. 1 week altitude or baseline).

(A) Females: One-way ANOVA showed a significant impact of treatment on creatine in the female PFC (F_2,26_ = 4.61, *p* = 0.02), STR (F_2,27_ = 7.29, *p* = 0.003), HIP (F_2,26_ = 9.37, *p* = 0.0009) and BST (F_2,26_ = 5.43, *p* = 0.01, Figure 9A). In post hoc tests, creatine levels were significantly higher with CRMH and CRMH + FLU vs. controls in the PFC, STR and BST, and with CRMH + FLU vs. controls in the HIP with (*p* < 0.05). When CRMH + FLU data were compared to 1 week creatine levels, Student’s *t*-test showed significantly higher creatine in the HIP with CRMH + FLU treatment vs. 1 week altitude (*p* = 0.01), but no difference was seen between the two groups in the PFC (*p* = 0.37), STR (*p* = 0.43) and BST (*p* = 0.84).

(B) Males: For males, one-way ANOVA showed a significant effect of treatment on creatine levels in the PFC (F_2,25_ = 3.47, *p* = 0.047), STR (F_2,18_ = 11.24, *p* = 0.0007), HIP (F_2,21_ = 6.84, *p* = 0.05) and BST (F_2,19_ = 8.4, *p* = 0.002, Figure 9B). In post hoc tests, creatine levels were significantly higher with CRMH + FLU vs. controls in all four brain regions, and with CRMH + FLU vs. CRMH in the STR and BST (*p* < 0.05). When CRMH + FLU data were compared to 1 week creatine levels, Student’s *t*-test showed no significant difference between the two groups in the PFC (*p* = 0.055), STR (*p* = 0.40), HIP (*p* = 0.29) or BST (*p* = 0.42).

#### 2.4.3. CRDS + SSRI Impact on Brain Serotonin

(A) Females: One-way ANOVA showed a significant impact of treatment on serotonin in the female PFC (F_2,39_ = 6.95, *p* = 0.003), STR (F_2,40_ = 5.46, *p* = 0.008) and the BST (F_2,42_ = 3.72, *p* = 0.03), but not the HIP (F_2,43_ = 0.474, *p* = 0.62, Figure 9C). In post hoc tests, serotonin levels in the female PFC and STR were significantly higher with CRMH and CRMH + FLU vs. controls (*p* < 0.05). For the female BST, serotonin in the CRMH + FLU group was significantly higher vs. controls.

(B) Males: For males, one-way ANOVA showed a significant effect of treatment on serotonin in the STR (F_2,32_ = 11.24, *p* = 0.0002) and HIP (F_2,32_ = 6.64, *p* = 0.004), but not in the PFC (F_2,37_ = 0.0018, *p* = 0.99) or BST (F_2,38_ = 0.375, *p* = 0.68, Figure 9D). For the male STR, post hoc tests found that CRMH + FLU significantly increased serotonin vs. both CRMH and controls.

## 3. Discussion

In this study, we document significant sex-based differences in impact of dietary CRMH on brain chemistry, depression-like behavior and SSRI efficacy at altitude. We found that endogenous brain creatine decreases significantly with time at moderate altitude in both sexes. Dietary CRMH treatment markedly improved brain bioenergetics in both sexes, increasing creatine levels in all four brain regions in females, and in the male STR and HIP. In females, dietary CRMH increased serotonin in the PFC and STR and improved depression-like behavior in the FST and SPT, with no change in motor function. In males at altitude, CRMH reduced brain serotonin in the STR and HIP and did not improve depression-like behavior. Surprisingly, combination treatment with CRMH and the SSRI fluoxetine (CRMH + FLU) improved the SSRI efficacy in males at altitude, but not in females. In males, CRMH + FLU significantly increased striatal creatine and serotonin in males at altitude vs. both controls and dietary CR. This is the first study to document an impact of CRMH treatment on brain serotonin.

Brain endogenous creatine decreases with time at altitude in both sexes. Live neuroimaging studies using proton magnetic resonance spectroscopy (proton-MRS) have similarly documented brain creatine deficits in both rodents and people at altitude. Forebrain creatine levels decreased significantly in female SD rats after a week at 10,000 ft [27], while hippocampal creatine levels decreased significantly in male SD rats after 48 h at 21,976 ft [26]. Human forebrain creatine levels were significantly lower in healthy long-term residents at 4500 ft vs. those at sea level [25]. Living at altitude may thus reduce the bioenergetic function in key brain regions involved in MDD. In a clinical study, acute exposure to hypoxia (10% oxygen, 40 min) significantly reduced cerebral creatine levels in healthy volunteers: total creatine (creatine + PCR) concentration directly correlated to that of arterial blood oxygen [43]. Reduced forebrain creatine is a marker for MDD [44,45]. lower forebrain creatine is seen in depressed patients vs. healthy controls [45], and effective treatment of MDD improved forebrain creatine levels [30,45]. Improving brain bioenergetics with CRMH may thus be a valid therapeutic approach for MDD, and especially for TRD at altitude and otherwise.

CRMH treatment impacted brain energetics in a distinct sex-based regional manner. Brain creatine levels increased two to three-fold with treatment in both sexes, to resolve the brain creatine deficit seen after 5 weeks at altitude in females, but not males (Figure 4). Previous studies show that CRMH treatment for 2–8 weeks caused an increase of 1.3-fold creatine in the whole brain in female rats [46]. Creatine obtained through dietary intake is continuously transported to the brain across the blood brain barrier via a specific creatine transporter (CRT) [44,47]. CRT activity regulates cellular creatine uptake, and CRT expression varies significantly between brain regions and cell types [44,47]. In male Wistar rats, CRT mRNA and protein are expressed at high levels in neurons in the cerebral cortex, HIP and cerebellum, and at lower levels in the STR and BST [48]. No data yet exists for female CRT expression. In our study, CRMH improved serum creatine levels seven-fold in females and 22-fold in males. Despite this, brain creatine improved in a more restricted fashion in males vs. females in our study, and this may reflect both differences in regional creatine transport and brain access to blood-borne creatine. Ninety percent of blood-borne creatine is shunted into skeletal muscle, with only a fraction accessible to the brain. Another reason for the sex difference in brain creatine uptake may thus be the larger body size and greater proportion of lean mass (and thus muscle mass) in male rats vs. females.

Dietary CRMH also altered brain serotonin levels in a sex-based manner. Serotonergic neurons are located in the raphe nuclei of the BST and regulate mood via projections to brain regions including the PFC, STR and HIP. The PFC and STR are critical in mood regulation, with serotonergic disruptions here implicated in MDD [49,50,51]. The HIP is involved in emotional and cognitive processing, which is altered in MDD [52]. Changes in basal serotonin levels in the PFC, STR and BST are directly linked to MDD [53]. Housing female rats at moderate altitude consistently reduces serotonin in the PFC, STR, HIP and BST [41], and CRMH treatment significantly improved serotonin levels in the PFC and STR (Figure 5). Male rats housed at 4500 ft exhibit a transient increase in serotonin in the HIP and BST at 2 weeks, but no change is seen with a longer duration of exposure [41]. In males at altitude, CRMH significantly reduced serotonin levels in the STR and HIP.

Dietary CRMH was antidepressant in the FST and reduced anhedonia in the SPT in females at altitude but was not effective in males at altitude (Figure 6 and Figure 7). CRMH did not alter motor function in either sex (Figure 6), which eliminates any impact of CRMH on motor activity, and validates increased immobility in the FST as a depression-like behavior. Our data are similar to that seen in preclinical dietary CRMH studies conducted at sea level with 2% *w/w* or 4% *w/w* CRMH for 5 weeks [38]. At sea level, dietary CRMH also did not alter motor function, and only the 4% *w/w* dose was effective at altering FST behavior. At sea level, 4% *w/w* CRMH decreased male swimming and LTI, and increased FST immobility [38], while at altitude, CRMH decreased male swimming but did not alter immobility or LTI (Figure 7). Dietary CRMH was thus not effective as an antidepressant in males at either sea level or altitude. In females at sea level, 4% *w/w* CRMH significantly increased swimming and LTI but did not change immobility in the FST [38]. In females at altitude, 4% *w/w* CRMH increased female swimming and LTI, and significantly decreased immobility (Figure 7). In female rats at sea level, dietary CRMH thus shows a trend to improving FST behavior [38], but at altitude, oral CRMH significantly reduced female depressive symptoms in the FST (Figure 7). Antidepressant effects seen in the rodent FST were confirmed in the SPT in the current study. Dietary CRMH increased sucrose consumption in females, thus reducing anhedonia in the SPT, while male SPT behavior did not change. Antidepressant effects of dietary CRMH in female rats correlated with improved brain energetics in MDD-linked brain regions, and increased serotonin levels in the PFC and STR. In males, dietary CRMH improved creatine levels, but not well enough to resolve the creatine deficit seen after housing for 5 weeks at altitude (Figure 1 and Figure 4). In males, dietary CRMH reduced brain serotonin levels and was not effective as an antidepressant in the FST or SPT. This is the first study to show a connection between dietary CRMH augmentation and brain serotonin.

In our model, rats housed at moderate altitude exhibited sex-based changes in depression-like behavior and brain biomarkers vs. those at sea level. Female rats exhibited a sustained increase in depression-like behavior in the FST and SPT with housing at altitudes of 4500 ft or 10,000 ft above sea level from 1–5 weeks, with no change in motor function in the OFT [40,41]. Females at moderate altitude also exhibit deficits in brain creatine and serotonin in MDD-linked brain regions vs. those at sea level [27,41]. In female rats, dietary CRMH thus resolves the altitude-related deficits in brain creatine and serotonin, and resolves altitude-related depression-like behavior in both the FST and SPT. After housing at moderate altitude for 2 weeks, male rats exhibit an increase in depression-like behavior in the FST and in brain serotonin vs. those at sea level [41], with altered motor behavior in the OFT, which may reflect changes in salience [41]. These changes were transient, however: brain serotonin, depressive symptoms and motor function did not differ between males at altitude vs. sea level with longer exposures [41]. However, like females, male rats also exhibit a sustained loss in the brain energetic marker creatine when housed at altitude. In males at altitude, dietary CRMH significantly improved brain creatine, but also reduced serotonin in the STR and HIP, and was not antidepressant in the FST or SPT.

In this model, both males and females also exhibit a sustained lack of antidepressant response to most SSRIs in the FST after housing at moderate altitude [42]. We found that the SSRIs fluoxetine (Prozac^®^), escitalopram (Lexapro^®^) and paroxetine (Paxil^®^) are effective antidepressants in the FST in both sexes housed at sea level. However, all three SSRIs fail as antidepressants in the FST in both sexes housed for 2 weeks at 4500 ft or 10,000 ft [41]. In the current study, fluoxetine was also not effective as an antidepressant in both sexes after 5 weeks at 4500 ft (Figure 8). However, when rats at altitude were given CRMH augmentation prior to SSRI treatment, we found a beneficial impact on SSRI efficacy in male rats but not females. In males at altitude, neither CRMH nor fluoxetine alone improved FST behavior, but CRMH + FLU showed antidepressant efficacy (Figure 8). In females at altitude, CRMH + FLU did not improve FST behavior. In studies carried out at sea level, sex-based effects of CRMH + FLU treatment varied markedly from that at altitude [39]. At sea level, CRMH + FLU treatment had no effect on FST behavior in male SD rats. However, in females at sea level, CRMH + FLU improved antidepressant efficacy of a sub-acute dose of fluoxetine (5 mg/kg), increasing swimming and LTI and decreasing immobility in the FST [39]. No change was seen on an effective dose of fluoxetine (10 mg/kg) [39]. In our previous studies [42] and the current one (Figure 8), fluoxetine at 20 mg/kg is similarly not an effective dose in either sex at altitude. Despite this, dietary CRMH markedly improved fluoxetine efficacy in only male SD rats at altitude, but not females. Sex-based impacts of dietary CRMH were thus distinctly different in SD rats housed at altitude vs. at sea level.

We further found that CRMH + SSRI treatment varied in impact on brain creatine and serotonin levels vs. dietary CRMH treatment alone. In males, CRMH + FLU treatment significantly increased creatine levels in all four brain regions vs. food controls, and in the STR and BST vs. dietary CRMH alone (Figure 9B). In the male STR, creatine levels increased two-fold with CRMH + FLU vs. CRMH, to resolve the creatine deficit seen vs. baseline. In males at altitude, CRMH + FLU also markedly increased serotonin in the STR vs. controls, while CRMH decreased striatal serotonin. In males at altitude, the antidepressant efficacy of CRMH + SSRI corresponded to improved STR serotonin. In females, creatine levels were higher with CRMH + FLU vs. CRMH only in the HIP but did not vary in other regions (Figure 9A). In females, brain serotonin was significantly higher with CRMH + FLU vs. controls in the PFC and BST but did not differ vs. CRMH in any region. Despite this, females on CRMH + SSRI exhibit a loss of the antidepressant efficacy shown with CRMH alone, suggesting that CRMH + FLU together may alter other aspects of brain chemistry over CRMH alone. Chronic hypoxia can also cause an imbalance in brain dopamine and norepinephrine levels [54], and our pilot data suggests that brain regional dopamine may increase in females with housing at altitude and be reduced in males [55]. SSRIs act by improving serotonergic transmission, but SSRI can also cause a hypo-dopaminergic state in humans and animal models [56]. In mice, chronic fluoxetine treatment (3 weeks, 10 mg/kg/day) reduces striatal dopamine and impairs motor function [57]. Further studies will assess whether CRMH + FLU alters brain dopamine or norepinephrine levels in rats at altitude.

A connection between brain energetics and serotonin levels has been previously shown. In male mice, treatment with the bioenergetic compound acetyl-L-carnitine improved ATP and PCR levels in the forebrain and other brain regions [58], and also improved brain serotonin and norepinephrine levels in the HIP and cerebral cortex. This study is the only other investigation connecting bioenergetic compounds to the enhancement of brain serotonin. However, a link is also documented between serotonergic antidepressants and brain energetic pathways. In male Wistar rats, chronic treatment with the SSRI paroxetine increased the activity of creatine kinase in the PFC, STR and HIP [59], while the serotonergic TCA imipramine increased creatine kinase activity in the PFC and HIP [60]. These data together suggest that significant crosstalk may exist between brain serotonergic and high energy metabolic pathways.

CR supplementation has been used for decades by athletes, with a good safety and tolerability profile [61]. MRS neuroimaging was used to evaluate changes in human brain chemistry which occur with CRMH treatment [33,34]. Consumption of oral CRMH (4 × 5 g per day, 4 weeks) increased whole brain creatine levels by 8.7% in six healthy volunteers [34] and this energetic change was reversable and no longer seen 12 weeks after treatment [34]. When healthy volunteers were given oral CRMH for 2 weeks (week1—0.3 g/kg/day, week2—0.03 g/kg/day) vs. placebo, the CRMH group showed a significant increase of 8.1–9.3% in brain total creatine levels and a marginal increase (3.4%) in PCR levels vs. controls [33]. These studies confirm the potential of using CRMH treatment to improve brain energetics in people with disorders linked to brain hypometabolism, such as MDD [33,34,62].

Dietary CRMH has been tested clinically as an augmentation treatment in women with MDD. Dietary CRMH (5 g/day, 8 weeks) improved the efficacy of the SSRI escitalopram (Lexapro^®^, 10–20 mg/day) in depressed women vs. SSRI alone [33]. In adolescent women with SSRI-resistant depression, oral CRMH (4 g/day, 8 weeks) significantly improved SSRI response and increased brain PCR levels vs. at baseline [36], with no changes seen in age-matched healthy controls over the 8 weeks. In a placebo-controlled study, adolescent women with SSRI-resistant MDD were given 2 g, 4 g or 10 g CRMH daily vs. placebo for 8 weeks along with prescribed SSRIs, and dietary CRMH augmentation significantly increased both forebrain PCR levels and improved MDD status vs. SSRI alone [37]. SSRIs used in these studies include fluoxetine, citalopram, escitalopram and sertraline at 40 mg/day [36,37]. MDD status improved from a week of treatment onwards, and frontal lobe PCR levels increased from 4.6% to 9% with oral CRMH [37]. Dietary CRMH can thus improve SSRI efficacy in female MDD patients but is yet to be tested clinically for antidepressant potential by itself.

Mode of action of dietary CRMH has been studied in other animal models of depression. In female mice, oral CRMH (1% or 10%, 3 weeks) was antidepressant in the tail suspension test, and increased levels of creatine kinase and brain derived neurotrophic factor (BDNF) mRNA and protein in the HIP [63]. Low levels of hippocampal BDNF are noted in animal models of stress-related depression, and hippocampal BDNF mRNA and protein levels also increase after treatment with classical antidepressants. In the HIP, CRMH increased mRNA of the anti-apoptotic proteins Bcl2 and Bcl-xL and reduced that of the pro-apoptotic Bcl2-associated death promoter (BAD) [63], implying that CRMH can protect against apoptosis. In a mouse model of corticosterone-induced depression, CRMH reversed the depressant effects of chronic corticosterone in the tail suspension test, by activation of mammalian target of rapamycin (mTOR) and phosphatidylinositol-3-kinase (PI3K) pathways [64]. CRMH also reversed the decrease in hippocampal BDNF levels caused by corticosterone, suggesting the CRMH may be effective in chronic stress-related depression. In rats, intra-hippocampal CRMH administration led to improvement in spatial memory [65], and this along with the dense hippocampal expression of creatine kinase suggests that creatine may play a critical role in learning and memory, which is altered in MDD. Of note, CRMH treatment, with and without SSRI, alters hippocampal serotonin levels significantly in both males and females at altitude (Figure 5 and Figure 9).

Brain creatine levels are decreased in rats exposed to hypobaric hypoxia in this study and others [26,27] in rats exposed to hypoxia/ischemia [66] as well as in healthy humans exposed to acute hypoxia (10% oxygen, 40 min) [43]. The high energy needs of the human brain suggest that even mild hypoxia can alter brain physiology. Creatine levels vary markedly by brain region, with regional levels corresponding to regional brain activity. In times of high energy demands or in hypometabolic conditions, such as hypoxia, the use of the CR/PCR pathway for rapid ATP synthesis may be critical for maintaining brain energetics at levels required for normal brain functioning [67]. Besides being involved in energetics, CRMH is also neuroprotective against hypoxia and oxidative stress. In a placebo-controlled dietary CRMH study, healthy adults exposed to hypoxia (10% oxygen, 90 min) showed cognitive deficits, which were reduced with CRMH treatment [68]. In in vitro studies, CRMH pre-treatment of rat hippocampal brain slices causes a decrease in the ischemic damage induced by transient anoxia [69,70]. Creatine pre-treatment also protects against transient cerebral hypoxia/ischemia in neonatal rat models, reducing hypoxia-induced seizures and death [61]. In preclinical studies, creatine interacts with the glutathione antioxidant pathway and can reduce the brain inflammatory response to hypoxia [71]. CRMH thus shows the ability to protect against hypoxia-induced damage to the brain in various paradigms.

Our data show that oral CRMH may be effective as an antidepressant at altitude (in hypobaric hypoxia), with or without SSRI treatment. In our model, CRMH shows antidepressant efficacy in SSRI-resistant depression at altitude. The hypobaric hypoxia experienced with living at altitude can cause deficits in blood oxygen levels and brain bioenergetic markers at as moderate an altitude as 4500 ft [23,25]. Living at altitude is linked to higher risk for MDD [4,5,6,7], and suicide [8,9,10,11,12,13,14] as well as for abuse of methamphetamine, cocaine and prescription opioids [72,73,74]. Both MDD and substance use disorders are linked to brain hypometabolic function and use of energetic compounds such as CRMH has been recommended as treatment for these metabolic disorders [33,34]. In clinical trials at altitude, CRMH improved depression status in both adolescent women with SSRI-resistant depression, and in adult women who use methamphetamine [36,37,75]. Our studies thus indicate that improving brain energetic function by treatment with bioenergetic compounds such as CRMH may be a valid approach to improving MDD status at altitude. The clinical implications of this study are that CRMH may be used as an add-on therapeutic for SSRI lack of efficacy at altitude or in TRD in general, or alternatively may be tested for antidepressant efficacy by itself in clinical trials. Our studies indicate that CRMH may be particularly effective for female depression.

### Limitations of Study

The following limitations apply to this study: (1) Estrous cycle staging was not conducted in this study, and the impact of CRMH at altitude may differ in females at different stages of the estrous cycle, as shown at sea level [38,39]. However, since our model is one of chronic hypoxic stress, we did not want females to experience any additional stressors vs. males, and therefore did not conduct estrous cycle staging. (2) We only tested single doses of both CRMH and the SSRI fluoxetine, chosen based on dose–response studies of CRMH previously tested in male and female rats at sea level [38,39], and dose–response for fluoxetine conducted at altitude [43]. (3) We found that CRMH improved creatine levels in brain regions linked to MDD. However, we did not assess whether this was a selective effect or whether CRMH treatment has a global impact on creatine levels in the brain in general, with potential implications for therapeutic strategies for other brain disorders. Despite these limitations, our studies indicate that CRMH treatment shows great potential as a therapeutic for treatment-resistant MDD, especially in women.

## 4. Materials and Methods

### 4.1. Animals

For all experiments, male and female Sprague Dawley rats of 125–150 g body weight were received from Charles River Laboratories (Raleigh, NC, USA). Rats were housed individually and given food and water ad libitum. Rats were randomly assigned to the different altitude groups. All animal studies were performed in accordance with the NIH Guide for Care and Use of Laboratory Animals. All animal procedures were approved by both the University of Utah Institutional Animal Care and Use Committees (IACUC, PHS Assurance #A3031-01, USDA #87-R-001, October 2014–September 2020) and the Veteran’s Administration Animal Component of Research Protocol (ACORP, VA Registration # A15/10, A18/16, September 2015–September 2021).

### 4.2. Study Groups

In study 1, endogenous brain creatine levels were assessed in rats after housing for 1–5 weeks at an altitude of 4500 ft. In our model, rats are acclimatized for a week at 4500 ft after delivery; thus, the baseline for study 1 is at 1 week of exposure to 4500 ft. In study 2, rats housed at 4500 ft were given dietary creatine monohydrate (CRMH) or normal food for 5 weeks, and then tested for (1) depression-like behavior in the FST, (2) anhedonia in the sucrose preference test (SPT), and (3) impact on brain creatine and serotonin levels. Another set of animals on dietary CRMH supplementation were tested for locomotor behavior in the open field test (OFT). In study 3, another set of animals were given dietary CRMH or normal food for 5 weeks, and then tested in the FST with SSRI treatment (CRDS + SSRI study), then assayed for brain creatine and serotonin. All studies were conducted in both male and female rats, and group sizes ranged from an *n* of 8–20.

### 4.3. Drug and Compounds

Creatine monohydrate (CR) was purchased as “Creapure” from AlzChem Inc., (Trostberg, Germany). The powdered rat chow used was Teklad Global T2020 rat diet meal from Envigo (Denver, CO, USA). Fluoxetine hydrochloride was received from NIMH Drug Supply Program.

### 4.4. Dietary Supplementation

Rats housed at local conditions of 4500 ft were given dietary supplementation with creatine monohydrate at 4% *w/w* in powdered food for 5 weeks as per [38,39], while control animals were given powdered food for 5 weeks. Dietary supplementation was set up within 24 h of delivery to our facility. Each rat was given 300 g food provided in disposable plastic feeder bowls stabilized with a rodent feeder shield (Unifab Inc, Kalamazoo, MI, USA), to prevent spillage [76]. At the end of each week, food bowls were weighed and replaced with a fresh 300 g supply of food. Food consumed and body weight were measured weekly, and the amount of CRMH consumed by each rat was calculated as the total gms of CRMH consumed per animal and gm/kg body weight per animal.

### 4.5. Forced Swim Test

After dietary treatment, depression-like behavior was measured in the modified FST [77], as previously described [40,78,79]. The FST was conducted in 2 sessions: the pretest session (15 min) followed 24 h later by the test FST (5 min) to assay depressive symptoms. In the FST, a rat was placed in a clear tank (25 cm wide, 65 cm tall) filled with 48 cm deep water at 25 °C, and its behavior was recorded. FST was conducted in ambient light at the same time of day (1–3 pm), with animals visually blocked from experimenters by a screen [79,80].

FST behavior was scored manually by investigators blinded to the experimental group. The time sampling technique of Detke [77,79] was used to score FST behavior. Behavior in the FST was scored as swimming (horizontal movements), climbing or diving (vertical movements) and immobility (passive floating) [77]. Time spent swimming, climbing or immobile was presented as a percent of total time. The latency to immobility (LTI) was calculated as the time taken to reach the first 10 s period of immobility [77,78,79]. Antidepressant efficacy in the FST was measured as a minimum 20% reduction in immobility vs. controls [78,80], and FST behavior was compared with that of the positive control, desipramine [42]. For negative controls, since FST behavior of the food control group did not vary from that of the saline control group used for antidepressant tests; these groups were combined to form the negative control group referred to as control.

### 4.6. Antidepressant Treatment

For study 2 and study 3, antidepressant treatment in the FST was conducted as per [42,81], with rats given 3 injections of antidepressant at 1 h, 19 h and 23 h after the pretest FST session. For study 2, the tricyclic antidepressant (TCA) desipramine (10 mg/kg) was used as a positive control. For study 3, rats in the CRMH and food groups were tested in the FST after treatment with the SSRI fluoxetine (20 mg/kg). Both antidepressants were made up in 0.9% saline and administered subcutaneously in a volume equivalent to 4 μL/gm body weight.

### 4.7. Sucrose Preference Test

The SPT is a two-bottle preference test for anhedonia, which was performed 24 h after the test FST session. Rats were given 2 bottles, one with water and the other with a 1% sucrose solution [82], and sucrose preference was measured. Bottles were switched at the 30 min time point to prevent side bias. Sucrose and water intake are assessed by weighing bottles before and after a 24 h period exposure to the two-bottle paradigm. Sucrose preference was calculated as the amount of sucrose solution consumed vs. total liquid consumed (water + sucrose solution).

### 4.8. Open Field Test

A separate set of animals were tested in the OFT after dietary CRMH treatment. The OFT was used to measure locomotor effects of dietary CRMH, towards eliminating the potential of motor activation effects of treatment and validating depressive symptoms in the FST as per [79]. For the OFT, a rat was placed in the center of the OFT box and its behavior was recorded as it moved freely for 10 m, as per [40,83]. Locomotor behavior in the OFT was analyzed with Noldus Ethovision software (Leesburg, VA) and measured as the distance covered and time spent moving vs. immobile for each animal.

### 4.9. Dissections

Rats given CRMH supplementation vs. food, then tested in the FST and SPT, were finally sacrificed to collect brain and blood samples. Rats were sacrificed 24 h after the SPT, brains rapidly isolated [84], and the prefrontal cortex (PFC), striatum (STR), hippocampus (HIP) and brainstem (BST) were dissected out. Coronal slices of the rat brain were cut of regions of interest, using a brain slicing mold (Ted Pella, Redding, CA, USA) and stereotaxic coordinates from [85]. The first cut was made at the optic chiasm (approx. Bregma −0.92), and the second cut was 3 mm rostral to this, separating the rostral-most slice (for the PFC) from the second (for the STR). The third cut made 6 mm below the first separates out the slice with the HIP, and the fourth cut made 5 mm below the third cut isolates the BST region. The PFC is dissected out from first slice as the rostral-most gray matter. The STR and HIP are dissected from the second and third slices, based on distinctive morphological appearance [84]. The BST is dissected from the caudal-most slice with removal of the visually defined pons and cerebellum. Brain tissue was flash-frozen on dry ice and stored at −80 °C until analysis. Blood was collected by cardiac puncture post-mortem, and serum extracted by centrifuging blood at 5000 G for 10 m at 4 °C. Serum was stored at −80 °C till analysis. Dissections were conducted from 8a.m.–12p.m., to avoid diurnal variation [86].

### 4.10. Tissue Creatine Analysis

Brain tissue was homogenized and then centrifuged at 14,000× *g* for 20 min using 10 Kd MW spin columns (Bio Vision, Milpitas, CA, USA) to remove excess protein. Serum was directly centrifuged in spin columns. The creatine content was measured in the supernatant using the Creatine Colorimetric/Fluorometric Assay kit (BioVision, Milpitas, CA, USA) as per instructions. The pellet was assayed for total protein using the Pierce^TM^ BCA Protein Assay Kit (Thermo Fisher Scientific, Rockford, IL, USA) to normalize to protein content for each sample. The optical density of CRMH samples was read at 570 nm and BCA samples optical density read at 562 nm. The total creatine per sample was calculated as nM CRMH normalized to sample protein content.

### 4.11. Brain Serotonin Analysis

Serotonin levels were measured in brain regions using the Serotonin Research ELISA kit from Rocky Mountain Diagnostics (Colorado Springs, CO, USA), which allows detection down to 0.005 ng/mg tissue. ELISA plates were read at 450 nm (BioTek Instruments, Winooski, VT, USA). The serotonin content of samples was extrapolated by comparing the optical density of samples (mean of duplicates) to that of the standard curve, using GraphPad Prism-9 software (GraphPad Software, San Diego, CA, USA). Data were normalized for weight of sample.

### 4.12. Statistical Analyses

Data were analyzed separately by sex and brain region. Statistical analyses were performed using GraphPad Prism-9 (GraphPad Software, San Diego, CA, USA). Data points more than 1.5 interquartile ranges below the first quartile or above the third quartile were treated as statistical outliers and removed from analysis. Data were analyzed by Student’s *t*-test for dietary CRMH vs. food groups, one-way ANOVA or two-way ANOVA. Data is presented as mean ± standard error of the mean (Mean ± SEM) and significance was determined by post hoc analyses using Tukey tests (*p* < 0.05).

## 5. Conclusions

These studies show that dietary CRMH supplementation can improve brain bioenergetic deficits at altitude in rats of both sexes, and has sex-based impacts on regional brain serotonin levels and antidepressant efficacy. In female rats, CRMH treatment corrects serotonin deficits at altitude and has antidepressant efficacy without any motor effects. In male rats at altitude, CRMH alone is not antidepressant, but CRMH combined with the SSRI fluoxetine improves brain serotonin levels and is antidepressant. Antidepressant efficacy of dietary CRMH or CRMH + SSRI are seen in parallel to improved serotonin levels in MDD-linked brain regions.

## Data Availability

Not applicable.
